# Unlocking the potential of multidisciplinary clinics to transform rare epilepsies care, insights, and research

**DOI:** 10.3389/fneur.2026.1619219

**Published:** 2026-02-12

**Authors:** Carole Bakhos, Christal G. Delagrammatikas, Scott Demarest, Yssa DeWoody, Tuesdi Dyer, Ilene Penn Miller, Amanda Moore, Ashley Fortney Point, Charlene Son Rigby, Jackie Steinberg, Vanessa Vogel-Farley, Kimberly Wiltrout

**Affiliations:** 1 Jordan’s Guardian Angels, Sacramento, CA, United States; 2 Malan Syndrome Foundation, Old Bridge, NJ, United States; 3 Department of Pediatrics and Neurology, Precision Medicine Institute, Children’s Hospital of Colorado, University of Colorado, Aurora, CO, United States; 4 Ring14, USA, Midland, TX, United States; 5 Cardio-Facio-Cutaneous Syndrome & RASopathies Network, Lake Worth, TX, United States; 6 Rare Epilepsy Network, Bethesda, MD, United States; 7 Angelman Syndrome Foundation, Aurora, IL, United States; 8 Koolen-de Vries Syndrome Foundation, Wilmington, NC, United States; 9 STXBP1 Foundation, Holly Springs, NC, United States; 10 Global Genes, Aliso Viejo, CA, United States; 11 Rare Epilepsy Network, Cedarburg, WI, United States; 12 Boston’s Children's Hospital, Boston, MA, United States

**Keywords:** Developmental and Epileptic Encephalopathies (DEEs), care coordination, clinical research and trials, integrated care models, medically complex care, multidisciplinary clinics, pediatric epilepsy, rare epilepsies

## Abstract

**Importance:**

Multidisciplinary clinics (MDCs) improve care for patients with complex, comorbid conditions through coordinated, team-based care. Despite their potential, MDCs remain underutilized and understudied in pediatric neurology, particularly for individuals with rare, chronic epilepsies.

**Observations:**

The subject of MDCs in pediatric epilepsy was explored through two workshops and surveys of caregivers and clinicians. MDC models vary widely—from general clinics (e.g., neurology, genetics, and neuropsychology) to disorder-specific clinics with multisystemic specialists. Caregivers identified key barriers, including geographical distance, personal expense, and insurance prior authorization requirements, yet overall reported positive experiences—citing valuable opportunities to participate in research and meaningful changes to clinical care. Although the findings reflect responses from a predominantly white, higher-income, English-speaking group of caregivers recruited through patient advocacy networks—and may therefore carry certain biases—their perspectives remain broadly generalizable to prospective patients across diverse socioeconomic settings. Similarly, physicians identified funding and space as the primary barriers to establishing multidisciplinary clinics, yet a majority recognized the importance of advancing research, translational studies, and clinical trials.

**Conclusions and relevance:**

MDCs can improve care for patients with medically complex rare epilepsies by integrating the management of comorbidities. These clinics bring value to both rare patients and physicians by providing a setting for synergistic activities between clinical care, clinical trials, and research. To expand their impact, we recommend: (1) establishing more MDCs using sustainable models; (2) improving access to extend the reach of MDCs; (3) including key specialists for integrated care; (4) sharing disorder-specific expertise through collaboration and training; and (5) tracking standardized success measures to validate and scale these efforts.

## 1 Introduction

Although individual underlying causes of epilepsy may be rare, collectively they represent an increasingly significant proportion of epilepsy cases, especially in the pediatric population (1, 2). Many individuals living with rare epilepsies are medically complex, with a prevalence of multisystemic comorbidities and in 30% of patients (3). Regardless of seizure control, comorbidities negatively impact quality of life, and these effects often worsen with age. A lack of coordination and communication between specialists perpetuates healthcare fragmentation, cost inefficiencies, and poor outcomes (4). A proposed solution is multidisciplinary care clinics (5), where specialists work together to deliver tailored, comprehensive, and coordinated care. The Rare Epilepsy Network (REN), a consortium of 170 Patient Advocacy Groups (PAG), led discussions at two multi-stakeholder workshops of caregivers, advocates, clinicians, researchers, and industry, with findings underscoring the significant value of multidisciplinary clinics (MDCs) as described both by patients who have attended or wish to attend them and by clinicians who direct or aspire to direct them.

## 2 Rare epilepsy network (REN) leads multidisciplinary care initiative (2021–2023)

A 2021 REN Member focus group identified a major gap in treatment for many rare epilepsy disorders. Multidisciplinary clinics (MDCs) offer comprehensive care by uniting specialists to manage comorbidities, improve care, and advance research. Disorders without access to such clinics require families to navigate the healthcare system on their own, piecing together care plans.

In 2021, REN’s annual workshop at the American Epilepsy Society (AES) conference focused on MDCs in rare epilepsies. The invite-only workshop consisted of 73 attendees including 27 PAGs, 16 Epilepsy Centers, six Young Investigators, six industry partners, and two representatives from the National Institute of Neurological Disorders & Stroke (NINDS) (see Supplementary Figure 1). Thought leaders Peter Davis, MD (Harvard Medical School) and Scott Demarest, MD, MSCS (Children’s Hospital Colorado) presented on Large Networked Single Syndrome Clinics and Small MDCs for Ultra Rare or Early-Stage Epilepsies, respectively. Attendees then broke into groups to discuss: MDC supports and obstacles; promising interventions; and successful models. All key stakeholders expressed support for MDCs. During the workshop, PAGs shared, “[h]aving a team that knows your child’s rare disease is priceless.” Clinicians advocated for, “Equity/access to care no matter where [patients] are located.” Further, they stated, “It feels fulfilling [to] learn from other colleagues across specialties.” Industry concluded that having “patients all in one place improves center readiness for clinical trials.”

Following the workshop, REN launched an MDC workgroup that developed a survey for clinicians and another for caregivers (see Supplementary material: Survey 1, Supplementary material: Survey 2). The study was reviewed by the Advarra Institutional Review Board (IRB), which determined that formal approval was not required (exempt status). The online surveys included multiple choice and free text. They were built in Google Forms and disseminated electronically via REN Members listserve, social media, newsletter, and monthly meetings, as well as partners (Epilepsy Foundation, CURE Epilepsy, Epilepsy Leadership Council, and AES). They included both qualitative and quantitative questions.

A second invite-only workshop was held during the 2022 American Epilepsy Society conference. The 101 attendees represented 29 PAGs, 13 Epilepsy Centers, 13 industry partners, the National Association of Epilepsy Centers (NAEC), and four Young Investigators (see Supplementary Figure 1). The REN working group presented the survey findings, which we have detailed below.

## 3 Caregiver and clinician perspectives on MDCs: key survey insights

### 3.1 Caregiver survey

The Caregiver survey resulted in 50 responses, predominantly from caregivers of minors (35) or adult children (7) affected by rare epilepsy. Most respondents were Caucasian (38), had college or advanced degrees (36), and came from households with incomes exceeding $100,000 (23). Their primary language was English (49) with most living in urban, suburban, or town centers (41). The persons living with epilepsy who are being cared for included 31 females and 19 males, aged from one to 51 years, representing 22 disorders, including many with Developmental and Epileptic Encephalopathies (DEEs).

Of the 50 respondents, half were unaware of a disorder-specific MDC for the disorder of the person with epilepsy. However, 23 of these 25 expressed interest in attending a non-specific MDC. The other 25 respondents had attended a disorder-specific MDC: 22 respondents learned about the existence of a clinic through a Patient Advocacy Group (PAG), 2 from a doctor, and 1 via an unspecified email. Analysis of MDC value and barriers is based on these 25 informed responses.

#### 3.1.1 Value of MDCs as described by caregivers

All 25 participants would recommend an MDC, and 24 (96%) found the visit was helpful and would return. Reported benefits fell into four categories: advanced expertise; better management; reduced stress and improved quality of life; and research opportunities.

- *Access to practitioners with advanced expertise and disorder-specific knowledge was highly valued (25/25)*. A caregiver survey responder commented, “*We learned from specialists familiar with her rare condition. None of our pre-existing medical team knew about her condition, and I do not believe she was being treated correctly.”* Caregivers reported helpful guidance on disease progression, managing expectations, and dealing with known comorbidities.
- *Many caregivers reported improved management through multi-specialist teams where clinicians collaboratively consider the whole patient rather than more narrowly through the lens of their individual specialty*. Of the 25 respondents, 14 (56%) noted improved care coordination and treatment adjustments, while 3 (12%) validated their existing care plan. One survey participant described cohesion across specialties, “You just feel like it is a complete puzzle as opposed to just talking piece by piece.” The most common changes in treatment approach resulting from attendance at an MDC included medication adjustments for seizure control, sleep, anxiety, and challenging behaviors, along with recommendations for behavioral interventions, new therapies, devices, and learning strategies. New diagnoses of autism, cortical visual impairment (CVI), and sleep disorders were noted that had not been made during standard medical care. The involvement of developmental pediatricians, neuropsychologists, and gastroenterologists was specifically mentioned.
- *Caregiver survey responders reported one-stop care with multi-specialty practitioners reduced stress on the family.* One participant expressed, *“So much less stress being able to see all [doctors] in one visit… [and doctors] who are familiar with my child’s medical condition and can collaborate…”*
- *Most participants 21/25 (84%) were offered and accepted research opportunities which they highly valued.* A caregiver responder shared, *“all participants are given the opportunity to consent into a research study. And I think everyone consents.”*

#### 3.1.2 Barriers to caregivers who participate in MDCs

Caregivers identified time, distance, hospital navigation, cost, and prior authorization as the primary challenges to participation in an MDC. Time was a significant issue, with 11 of 25 patients (44%) waiting 3 months or more for an appointment at an MDC. Attending an MDC often required substantial time away from home, work, and other children. Of the respondents, 16 of 25 (64%) were seen in a single day, while the rest required multiple days. Most participants faced long travel distances, with 11 participants traveling over 500 miles, made more challenging by their children’s complex needs. Financial burdens were considerable, with families reporting travel and accommodation costs between $1,000 and $2,500; two families incurred even higher expenses. Among 25 respondents, 12 (48%) had full insurance coverage; 12 (48%) had partial coverage; and one was denied any coverage. Caregivers also reported challenges with prior authorization.

### 3.2 Clinician survey

The Clinician Survey received 25 responses from 20 institutions offering different types of MDCs with some responders providing details on multiple clinics and two offering aspirational responses about the clinics they hoped to establish. These clinics spanned the United States but were concentrated in metropolitan areas (see Supplementary Figure 2).

The three types of MDCs surveyed included: *general neurology clinics* serving larger populations like genetics epilepsies, women, or surgery candidates (9 of 25); *clinics serving related disorders* like Dup15q/Angelman, RASopathies, or channelopathies (11 of 25); or clinics focused on a *single disorder* such as Rett Syndrome or Tuberous Sclerosis Complex (5 of 25). The general neurology clinics included: neurology (25 of 25), genetics (23 of 25), and neuropsychology/psychiatry (20 of 25). In contrast, disorder-specific clinics tended to include more specialists for comorbidities—providing coordination of multisystemic care (see Supplementary Table 1).

Clinics followed different models: 5 of 25 (20%) use an “open shop” model (patients attend multiple appointments and move rooms to meet with the different clinicians); 9 of 25 (36%) use a “flow shop” model (clinicians come to the patient’s room); and 10 of 25 (40%) use a “hybrid.” Seven clinics (28%) serve as the patient’s medical home, 10 (40%) act solely as consultants to local providers, and 8 (32%) provide both.

Despite 18 of 25 (72%) providing some level of consultancy, only 2 (8%) corresponded directly with local providers. 20 of 25 (80%) provided families with a written summary. Sixteen clinics (64%) held coordinated care meetings to reach a consensus on the patient’s care plan, 5 (20%) reported that such a meeting might happen, and 4 (16%) did not hold them. Thus, the level of coordination and comprehensiveness of the care plans varied by clinic.

#### 3.2.1 Value of MDCs as described by clinicians

All clinicians reported that the primary goal was to improve patient care. Of the 25, 20 (80%) were also committed to clinical and translational research with most disorder-specific clinics collecting natural history data. Clinicians highlighted the value of MDCs in improving care, providing access to experts, enhancing treatments and diagnosis, facilitating research, improving efficiencies for families, and building best practices. Clinician survey responders agreed that MDCs “crystallize best practices for complex disorders.” However, 17 of 25 (68%) reported that MDC success was not measured nor reported to their institutions. Clinicians internally tracked success based on patient volume, repeat visits, referrals, improved outcomes, family satisfaction surveys, research synergies (including grants, publications, and clinical trials), enhanced communication among stakeholders, and ongoing financial support from PAGs.

#### 3.2.2 Barriers to establishment and maintenance of MDCs as described by clinicians

**Figure 1:**
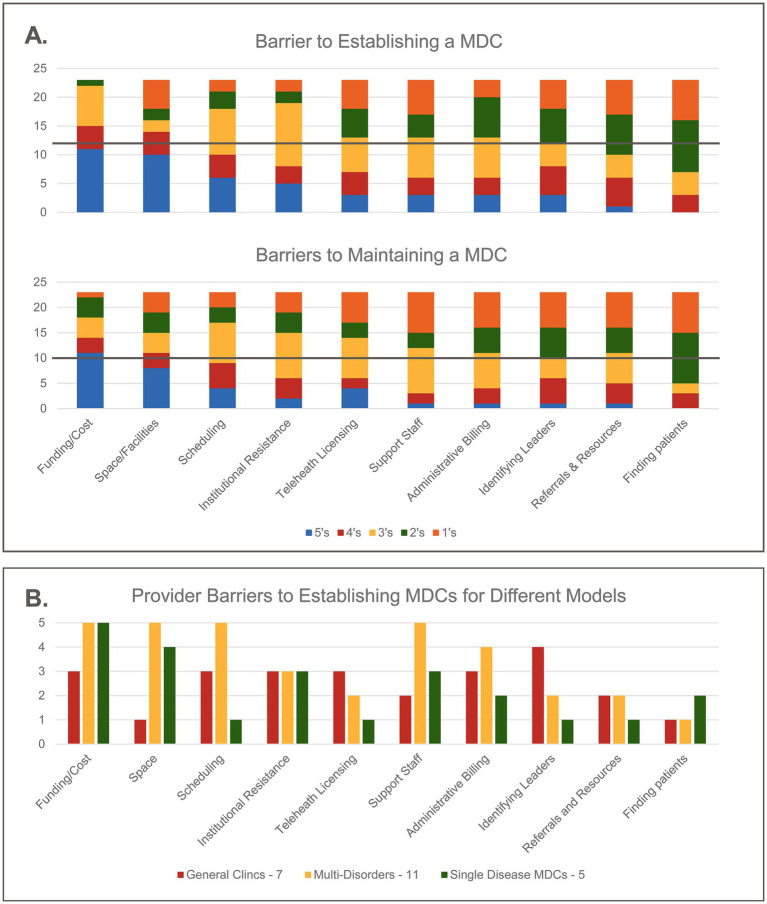
**(A)** Clinicians were asked to rank barriers to establishing an MDC (top graph) and to maintaining an MDC (bottom graph) from 1 to 5 with 1 being the lowest barrier and 5 being the highest barrier. These graphs show the distribution of the ranks and the line denotes the median rank for each barrier. **(B)** The same data set from barriers to establishing an MDC were grouped into types of clinics: General (*n* = 7), multi-disorder specific (*n* = 11), and single disorder (*n* = 5) (note: The two aspirational entries were not included). The bars denote the most common rank of each barrier within each group (i.e., the mode rank).

Barriers to establishing and maintaining an MDC were identified at the first REN workshop. To characterize the effort required to establish and then maintain an MDC, surveyed clinicians were asked to rank these barriers from 1 to 5 (1 being the lowest barrier and 5 being the highest barrier) (see Figure 1. Distribution of the rank of barriers associated with establishing or maintaining an MDC).

When all clinics were grouped together key findings indicate that funding and space were the highest barriers for both establishing and maintaining MDCs—the most common rank for both barriers was 5. The overall burden for establishing an MDC was not significantly different than maintaining an MDCs implying that these barriers may not lessen with time. Finding patients was the lowest barrier for both establishing and maintaining MDCs with rank 2 representing the median and mode of both distributions.

When clinics were grouped according to general versus multi-disorder versus single disorder clinics, barriers of funding and space emerged highest for both disorder-specific and single disorder clinics, with these barriers being lower for general clinics. Finding patients was identified as the lowest barrier for general and multi-disorder clinics, while single disorder clinics ranked identifying leaders, telehealth licensing, and referrals and resources as their lowest burdens. The distributions of the ranks for many of the barriers is almost uniform suggesting that each clinic is somewhat unique in the challenges they face.

Given that institutional support was a significant barrier, clinicians were asked to define institutional responsibilities to the MDC. Among the 17 responses, several key themes emerged: institutions should provide infrastructure, including space, billing support, clinical and behavioral health resources, and non-reimbursable support staff, e.g., nurse coordinator.

## 4 Discussion and recommendations

It should be noted that both surveys displayed some inherent biases in the responses. Caregiver survey respondents were predominantly educated, affluent, Caucasian families with insurance that they deemed as good. This bias mirrors a known bias in access to higher-level care (6, 7). Nevertheless, our findings represent a good initial assessment of MDCs that may be further assessed in future research for generalizabilty to those with different socioeconomic and racial backgrounds. Although the findings are U.S.-centric, the underlying concepts have broad international relevance and resonance. (8, 9).

Nearly all caregivers were involved with disorder—specific support groups and half had attended disorder-specific MDCs. Most responding clinicians were also engaged with disorder-specific organizations and research, indicating their sympathy towards the inherent challenges of caring for these complex communities. This resulted in a bias favoring disorder-specific MDCs in the responses.

Caregivers and clinicians agreed that MDC participation led to more comprehensive care plans, timely access to disease experts and specialists, research opportunities, and overall satisfaction. Despite barriers identified by caregivers (time, distance, expense) and providers (funding, space), there was strong support for more MDCs. It was surprising that, given the critical role institutions play in supporting MDCs, there was no standard for assessing the success or formally reporting back to the institute. Using standardized measures to quantify a clinic’s value would help secure more support and will be addressed in our recommendations.

Collaboration of clinicians across multiple disciplines benefits the patients, improves health outcomes, and advances our understanding of disorders—this has been demonstrated across many complex diseases including epilepsy (5), inherited metabolic diseases with epilepsy (10), Prader-Willi syndrome (11), Duchenne Muscular Dystrophy (12), movement disorders (13), and more. Furthermore, the National Association of Epilepsy Centers (NAEC) endorsed multidisciplinary care in their 2023 guidelines (14). Like advancements in oncology care, the care of individuals with epilepsy, particularly rare and severe forms, will be enhanced by teams that include clinicians, mental health advocates, social services, and specialized care providers. Towards this path, we make the following recommendations:

- Authors’ recommendations for establishing more MDCs using sustainable models

**Figure 2:**
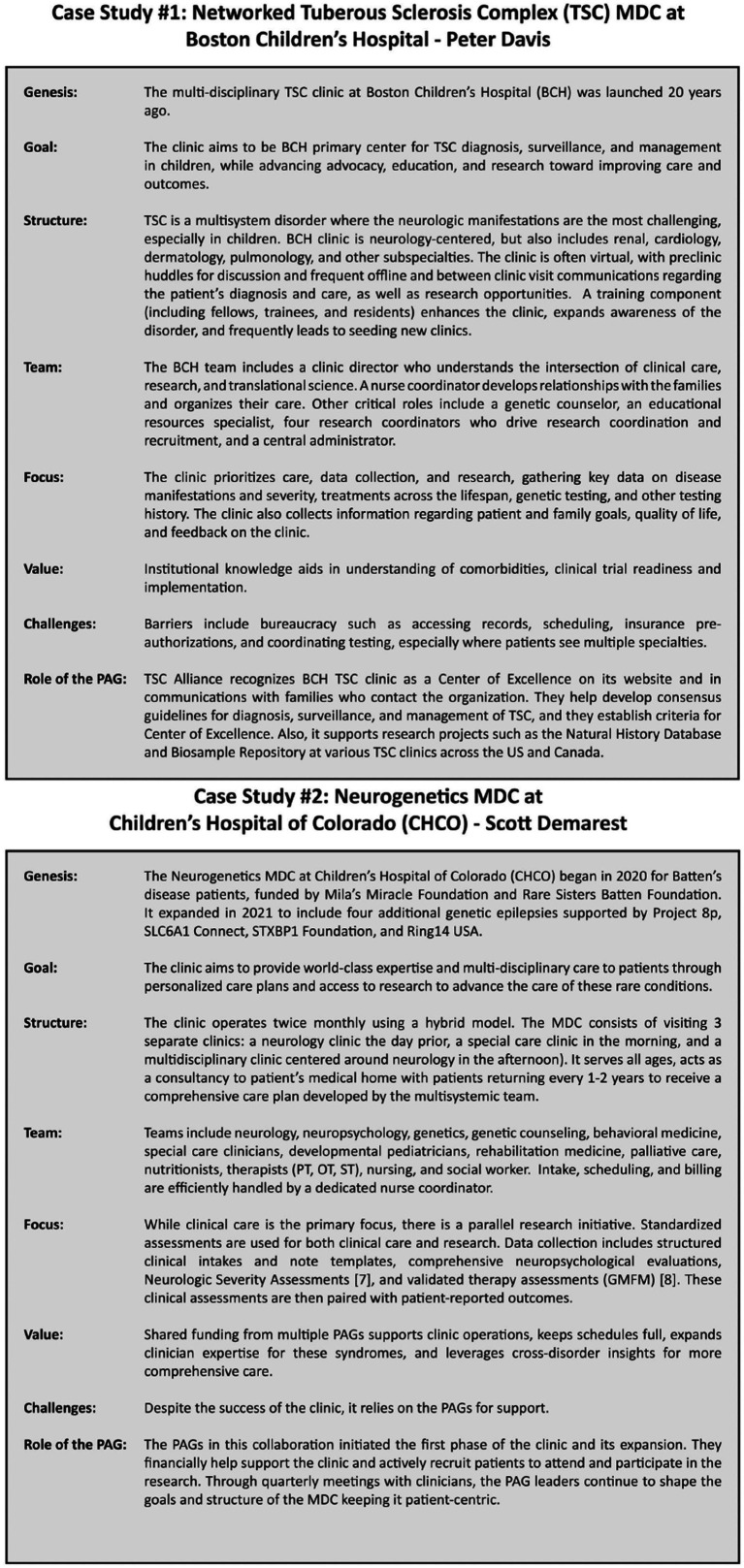
Case studies were presented during REN’s 2021 Workshop. Case study 1 presented by Peter Davis, MD, for networked multidisciplinary clinic for tuberous sclerosis complex at Boston Children’s Hospital. Case study 2 presented by Scott Demarest, MD, for a neurogenetics multidisciplinary clinic at Children’s Hospital of Colorado (CHCO).

We urge institutions to collectively conduct a needs-based assessment to address wait lists as well as geographic and disorder expertise gaps in MDC coverage. This could be done in collaboration with the National Association of Epilepsy Centers (NAEC) or Pediatric Epilepsy Research Consortium (PERC). In our case studies, Figure 2, we featured two MDC models as examples: (1) a networked MDC for TSC patients, and (2) a neurogenetics MDC that serves multiple disorders in a collaborative system. These are just two examples of the multiple MDCs that were examined in the clinician surveys.

Recently, additional clinics have been launched—often championed by PAGs—including clinics focused on Multidisciplinary Center for CACNA1A-Related Disorders (15) and Multidisciplinary Center for SCN2A-Related Disorders at UT Health (16), Malan Syndrome as part of the Beckwith-Wiedmann Syndrome Clinic at Children’s Hospital of Philadelphia (CHOP) (17), and a SCN8A/SCN2A Multidisciplinary Center at Children’s Hospital Colorado (18). These represent meaningful progress for our community.

At the same time, there remains a dearth of MDCs for most rare epilepsy disorders. Given the barriers to establishing and maintaining MDCs—particularly those that are disorder-specific—a broader, more flexible model may be necessary for long-term sustainability. Clinics need not be disorder-specific but must be disorder-knowledgeable. Expanding targeted clinics in underserved areas and addressing geographic or expertise gaps would redress the increasing demand and improve access to specialized care.

- Improve access to and extend reach of MDCs

Enhancing care delivery involves both setting up additional clinics based on strategic geographic and expertise gaps, as well as broadening the accessibility and outreach of existing ones. While neurologists are encouraged to refer refractory epilepsy patients to general epilepsy clinics to improve seizure control and diagnosis (5), PAGS are often leading the efforts in spreading awareness to patients regarding disorder specific MDCs. Understanding barriers to and incentivizing clinician referrals should be explored. To improve connectivity, REN recently launched a searchable list of existing MDCs that include specialists by area of clinical expertise and research (19). These recommendations, focused on pediatric rare epilepsy populations, are also applicable to adults (20).

Regulatory changes will also be important toward improving equitable access to MDCs. Encouraging payors to cover expenses for family travel to MDCs and accepting and expediting Medicaid coverage across state lines would extend access to care. Permitting same-specialty consults on the same day would reduce family burden. (21) Additionally, overcoming state-centric insurance and licensure barriers—many of which were waived during COVID-19—remains a critical step (22).

Additionally, for medically fragile and complex children, extending telehealth access would expedite access, reduce travel time, and eliminate many of the physical, emotional, and financial burdens on families. Successful telehealth models exist for some rare epilepsies (23), other neuromuscular disorders (24), and stroke (25). Facilitating primary doctor-to-specialist consultations will improve understanding of these disorders and enhance localized care.

- Include key specialists and coordinated care of comorbidities

Greater Access to multidisciplinary and multisystemic specialties to address comorbidities collaboratively enhances care and reduces parental stress. Key specialists may differ depending on the goal of the MDC and the patient population; however, neurologists, genetic counselors, geneticists, and neuropsychologists were found to play a key role in those we surveyed (26).

As our community considers other models of multidisciplinary care across ages, lifespan clinics may be another viable alternative (27). These clinics—offering continuous care from childhood through adulthood—recognize the challenges in transitioning complex disorders from pediatric to adult care.

- Leverage disorder-specific expertise through training, collaboration, and open-access

MDCs advance the development of clinical and institutional expertise, as well as evidence-based standards of care, which are severely limited within rare epilepsies (27). TeleECHOs, such as the (Not So Rare) Epilepsies ECHO series launched in 2023 and 2025 (28), offer a valuable way to share expertise from specialists with each other as well as frontline care providers. New research mechanisms that incentivize cross-institute collaboration and sharing will be key to extending learnings across centers.

Beyond care, MDCs serve a critical role in advancing data collection and research that would not otherwise occur because rare populations are too small, dispersed, and diffuse. Therefore, we urge increased access to data to accelerate and enhance synergistic activities between clinical care, clinical trials, data collection, and research. Learning Health Systems as exemplified by Epilepsy Learning Health System (ELHS) (29) and Pediatric Learning Health System (PELHS), (30) as well as the Pediatric Epilepsy Research Consortium (PERC) (31) are promising examples of existing cross-institute research and care collaborations that would benefit from additional incentives and resources.

- Develop and share standardized metrics demonstrating the institutional value of MDCS

MDCs enhance institutional prestige, and early investment in their development can attract additional funding and long-term support. Robust standardized metrics are essential for sustaining institutional investment and independently validating the clinical and operational value of MDCs. Therefore, we urge the development, implementation, and tracking of objective measures for the success of MDCs.

Key measures could include volume and reimbursements but should also track improved patient outcomes and quality of life, healthcare cost-savings (e.g., fewer ER visits and hospitalizations) and improved access for diverse populations. Peer-reviewed publications, federally awarded funding, novel clinical trials, and PAG research grants might also be considered indicators of success. Patient and caregiver satisfaction should be tracked and prioritized. Consistent reporting of these standardized success metrics to institutions will help to overcome many of the barriers to establishing and maintaining MDCs.

## 5 Conclusion

There is no one-size-fits-all model for multidisciplinary clinics (MDCs) serving the rare epilepsy community, yet numerous examples exist that can be adopted or adapted to local contexts. By accumulating patient cohorts, future MDCs are poised to lead precision medicine efforts, offering more personalized care and facilitating patient stratification for clinical trials (32, 33). Although our findings are limited by sample size and potential bias, they provide important initial evidence that MDCs deliver tangible benefits for both patients and clinicians, which can be further elaborated for broader populations in future studies. Each institution and motivated clinician should evaluate what works best given their location, resources, staff, and patient population. At the same time, the growing number of newly diagnosed families underscores the urgent need to expand MDCs strategically. Ensuring broad and equitable access—across geographic regions and rare epilepsy types—must remain a priority to meet this rising demand and deliver high-quality care for all.

## Data Availability

The raw data supporting the conclusions of this article will be made available by the authors, without undue reservation.

## Supplementary material

SUPPLEMENTARY FIGURE 1 MDC Workshop Stakeholders 2021 & 2022.

SUPPLEMENTARY FIGURE 2 MDC clinics as reported by Caregivers, Clinicians or both.

## References

[1] Ruggiero SM Xian J Helbig I. The current landscape of epilepsy genetics: where are we, and where are we going? Curr Opin Neurol. (2023) 36:86–94. doi: 10.1097/WCO.0000000000001141, 36762645 PMC10088099

[2] Oliver KL Scheffer IE Bennett MF Grinton BE Bahlo M Berkovic SF. Genes4Epilepsy: an epilepsy gene resource. Epilepsia. (2023) 64:1368–75. doi: 10.1111/epi.17547, 36808730 PMC10952165

[3] Ho NT Kroner B Grinspan Z Fureman B Farrell K Zhang J . Comorbidities of rare epilepsies: results from the rare epilepsy network. J Pediatr. (2018) 203:249–258.e5. doi: 10.1016/j.jpeds.2018.07.0552, 30195559

[4] Council on Children with Disabilities and Medical Home Implementation Project Advisory Committee. Patient- and family-centered care coordination: a framework for integrating care for children and youth across multiple systems. Pediatrics. (2014) 133:e1451–60. doi: 10.1542/peds.2014-0318 24777209

[5] Stern J Stanton S Howe-Martin L Lane C Sports C Gidal B . The multidisciplinary team in the treatment of patients with epilepsy. Epilepsy Curr. (2024) 15357597241242250. [Epub ahead of print]. doi: 10.1177/15357597241242250 39554270 PMC11561941

[6] Miller JS Oladele F McAfee D Adereti CO Theodore WH Akinsoji EO. Disparities in epilepsy diagnosis and management in high-income countries: a review of the literature. Neurol Clin Pract. (2024) 14:e200259. doi: 10.1212/CPJ.0000000000200259, 38585438 PMC10996906

[7] Kroner BL Fahimi M Kenyon A Thurman DJ Gaillard WD. Racial and socioeconomic disparities in epilepsy in the District of Columbia. Epilepsy Res. (2013) 103:279–87. doi: 10.1016/j.eplepsyres.2012.07.005, 22858309 PMC4608437

[8] Riva A Coppola A Bisulli F Verrotti A Bagnasco I Elia M . Italian report on RARE epilepsies (i-RARE): a consensus on multidisciplinarity. Epilepsia Open. (2024) 9:1857–67. doi: 10.1002/epi4.13020, 39176980 PMC11450651

[9] von Stülpnagel C van Baalen A Borggraefe I Eschermann K Hartlieb T Kiwull L . Network for therapy in rare epilepsies (NETRE): lessons from the past 15 years. Front Neurol. (2021) 11:622510. doi: 10.3389/fneur.2020.622510, 33519703 PMC7840830

[10] Tumienė B del Toro Riera M Grikiniene J Samaitiene-Aleknienė R Praninskienė R Monavari AA . Multidisciplinary care of patients with inherited metabolic diseases and epilepsy: current perspectives. J Multidiscip Healthc. (2022) 15:553–66. (Published 2022 Mar 25). doi: 10.2147/JMDH.S251863 35387391 PMC8977775

[11] Duis J van Wattum PJ Scheimann A Salehi P Brokamp E Fairbrother L . A multidisciplinary approach to the clinical management of Prader-Willi syndrome. Mol Genet Genomic Med. (2019) 7:e514. doi: 10.1002/mgg3.514, 30697974 PMC6418440

[12] Bushby K Finkel R Birnkrant DJ Case LE Clemens PR Cripe L . Diagnosis and management of Duchenne muscular dystrophy, part 1: diagnosis, and pharmacological and psychosocial management. Lancet Neurol. (2010) 9:77–93. doi: 10.1016/S1474-4422(09)70271-6, 19945913

[13] van Egmond ME Eggink H Kuiper A Sival DA Verschuuren-Bemelmans CC Tijssen MAJ . Crossing barriers: a multidisciplinary approach to children and adults with young-onset movement disorders. J Clin Mov Disord. (2018) 5:3. Published 2018 Apr 6. doi: 10.1186/s40734-018-0070-x, 29636982 PMC5887190

[14] Lado FA Ahrens SM Riker E Muh CR Richardson RM Gray J . Guidelines for specialized epilepsy Centers: executive summary of the report of the National Association of epilepsy Centers guideline panel. Neurology. (2024) 102:e208087. doi: 10.1212/WNL.0000000000208087, 38306606 PMC10962912

[15] Multidisciplinary Center for CACNA1A-Related Disorders. Available online at: https://www.utphysicians.com/multidisciplinary-center-for-cacna1a-related-disorders/ (accessed April 22, 2025)

[16] Multidisciplinary Center for SCN2A-Related Disorders. Available online at: https://www.utphysicians.com/multidisciplinary-center-for-scn2a-related-disorders/ (accessed April 22, 2025)

[17] Beckwith-Wiedemann Syndrome Clinic. Available online at: https://www.chop.edu/centers-programs/beckwith-wiedemann-syndrome-clinic (accessed April 22, 2025)

[18] The Multidisciplinary Clinic on Anschutz Medical Campus. Available online at: https://www.childrenscolorado.org/locations/anschutz-medical-campus-aurora/resources/multidisciplinary-clinic/ (accessed April 22, 2025)

[19] Rare Epilepsy Network, Clinics for Rare Epilepsies. Available online at: https://www.rareepilepsynetwork.org/clinics-for-rare-epilepsies (accesses October 28, 2025)

[20] Scheffer I. Pre-recorded: developmental and epileptic encephalopathies—where to start with managing such complex adult patients. J Neurol Sci. (2023) 455. Available online at: https://www.jns-journal.com/article/S0022-510X(23)00478-1/fulltext

[21] Szaflarski JP Rackley AY Lindsell CJ Szaflarski M Yates SL. Seizure control in patients with epilepsy: the physician vs. medication factors. BMC Health Serv Res. (2008) 8:264. doi: 10.1186/1472-6963-8-264, 19094222 PMC2642800

[22] Nguyen AM Farnham JJ Ferrante JM. How COVID-19 emergency practitioner licensure impacted access to care: perceptions of local and national stakeholders. J Med Regul. (2022) 108:7–19. doi: 10.30770/2572-1852-108.4.7

[23] Kramer ZJ Brandt C Havens K Pasupuleti A Gaillard WD Schreiber JM. Telehealth for patients with rare epilepsies. Ther Adv Rare Dis. (2022) 3:26330040221076861(Published 2022 Mar 8). doi: 10.1177/26330040221076861, 37180417 PMC10032469

[24] Carroll K Adams J de Valle K Forbes R Kennedy RA Kornberg AJ . Delivering multidisciplinary neuromuscular care for children via telehealth. Muscle Nerve. (2022) 66:31–8. doi: 10.1002/mus.27557, 35426158 PMC9325549

[25] Sharrief AZ Guzik AK Jones E Okpala M Love MF Ranasinghe TIJ . Telehealth trials to address health equity in stroke survivors. Stroke. (2023) 54:396–406. doi: 10.1161/STROKEAHA.122.039566, 36689591 PMC11061884

[26] Goldstein J Plioplys S Zelko F Mass S Corns C Blaufuss R . Multidisciplinary approach to childhood epilepsy: exploring the scientific rationale and practical aspects of implementation. J Child Neurol. (2004) 19:362–78. doi: 10.1177/088307380401900509, 15224709

[27] Balestrini S Guerrini R Sisodiya SM. Rare and complex epilepsies from childhood to adulthood: requirements for separate management or scope for a lifespan holistic approach. Curr Neurol Neurosci Rep. (2021) 21:65. doi: 10.1007/s11910-021-01154-7, 34817708 PMC8613076

[28] Epilepsy Foundation, Epilepsies ECHO HUB. Available online at: https://www.epilepsy.com/programs/echo (accessed October 28, 2025)

[29] Donahue MA Herman ST Dass D Farrell K Kukla A Abend NS . Establishing a learning healthcare system to improve health outcomes for people with epilepsy. Epilepsy Behav. (2021) 117:107805. doi: 10.1016/j.yebeh.2021.107805, 33588319

[30] Shah PD Yun M Wu A Arnesen RA Storey M Sokoloff M . Pediatric Epilepsy Learning Healthcare System Quality of Life (PELHS-QOL-2): A novel health-related quality of life prompt for children with epilepsy. Epilepsia. (2022) 63:672–685. doi: 10.1111/epi.17156 34971001

[31] Pediatric Epilepsy Research Consortium (PERC). About Us. Available online at: https://www.perc-epilepsy.org/our-story (Accessed January 22, 2026).

[32] Wang S Perucca E Berkovic SF Perucca P. Precision therapies for genetic epilepsies in 2025: promises and pitfalls. Epilepsia Open. (2025). doi: 10.1002/epi4.70065 PMC13394099 40411479

[33] Smith LA Ullmann JF Olson HE Achkar CM Truglio G Kelly M . A model program for translational medicine in epilepsy genetics. J Child Neurol. (2017) 32:429–36. doi: 10.1177/0883073816685654, 28056630 PMC5625332

