# Supplementary figures and images for: Unlocking the potential of multidisciplinary clinics to transform rare epilepsies care, insights, and research
Source: Front Neurol. 2026 Feb 12;17:1619219. doi: 10.3389/fneur.2026.1619219 (PMC12935687; doi:10.3389/fneur.2026.1619219)

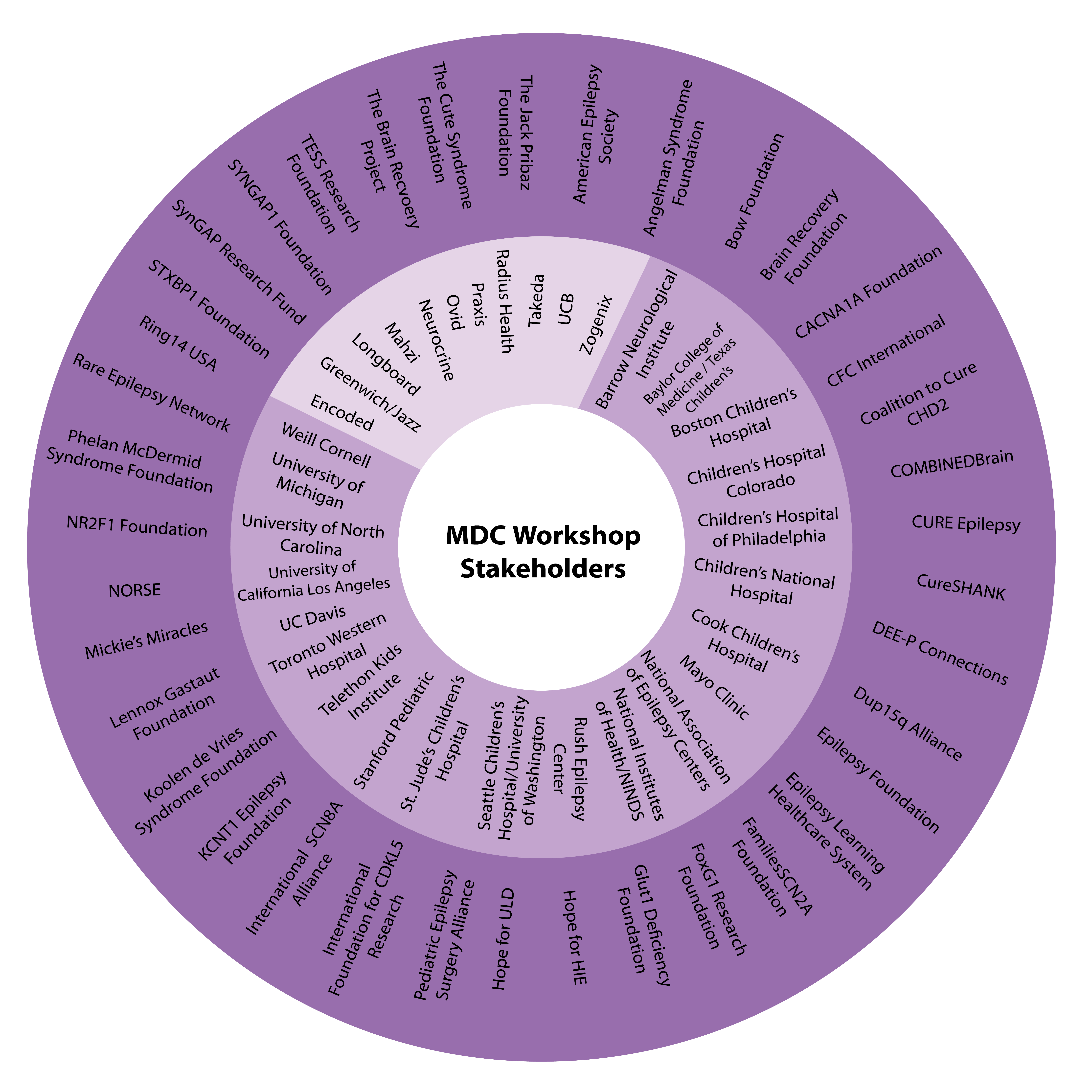

Supplement: SUPPLEMENTARY FIGURE 1 — MDC Workshop Stakeholders 2021 & 2022. [file Image_1.jpeg]

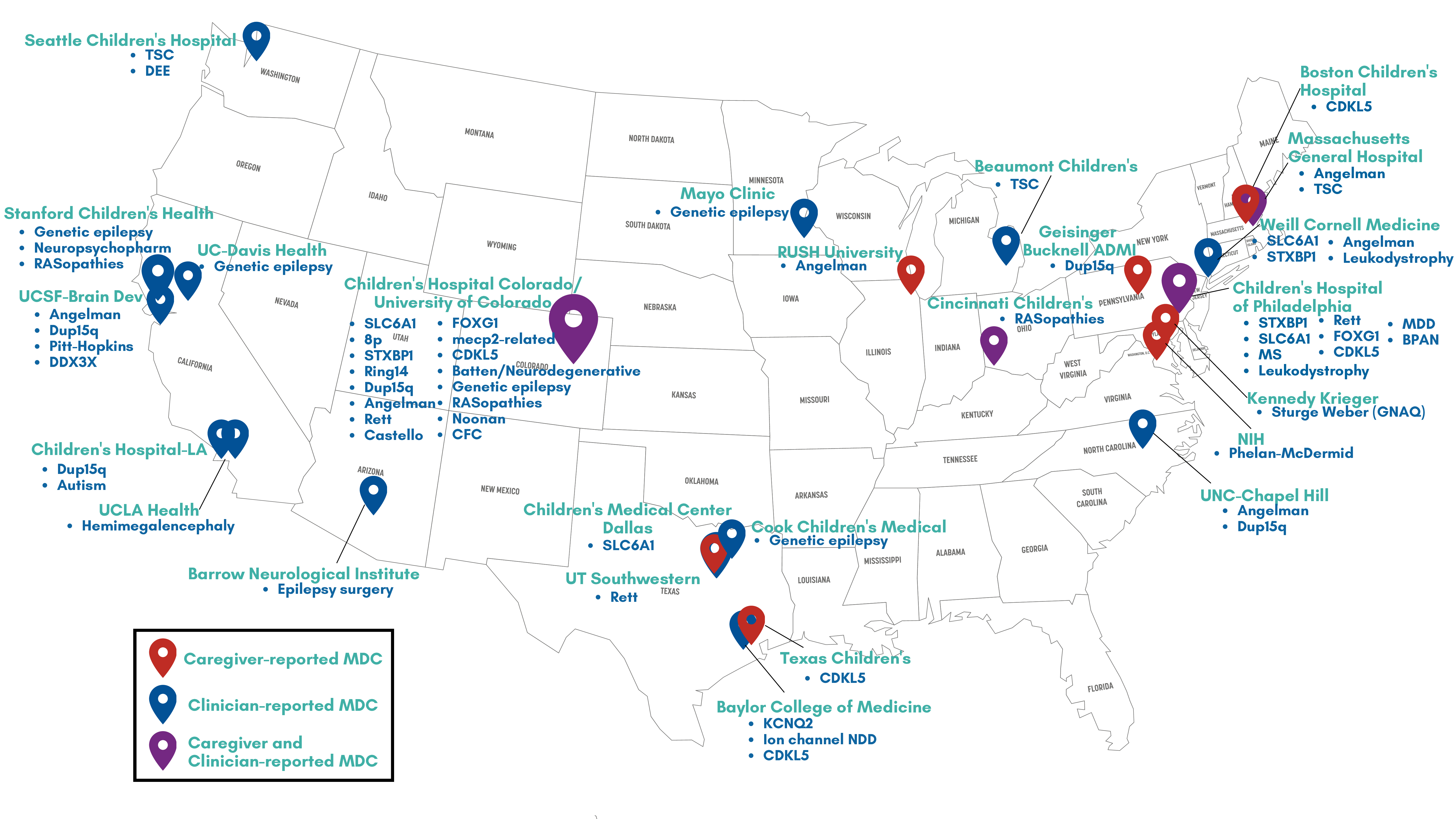

Supplement: SUPPLEMENTARY FIGURE 2 — MDC clinics as reported by Caregivers, Clinicians or both. [file Image_2.jpeg]

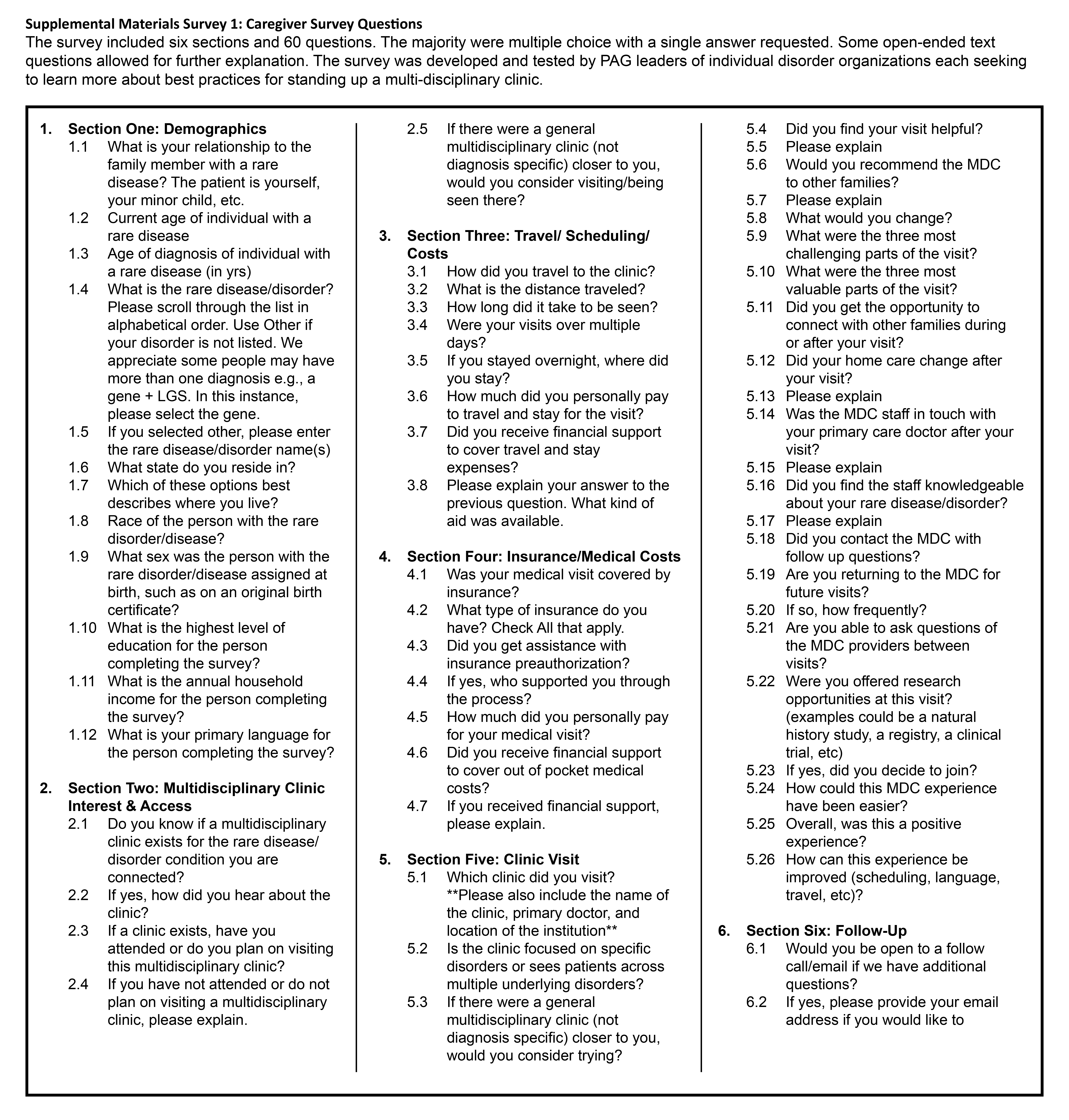

Supplement: Supplementary file 3 [file Supplementary_file_1.jpeg]

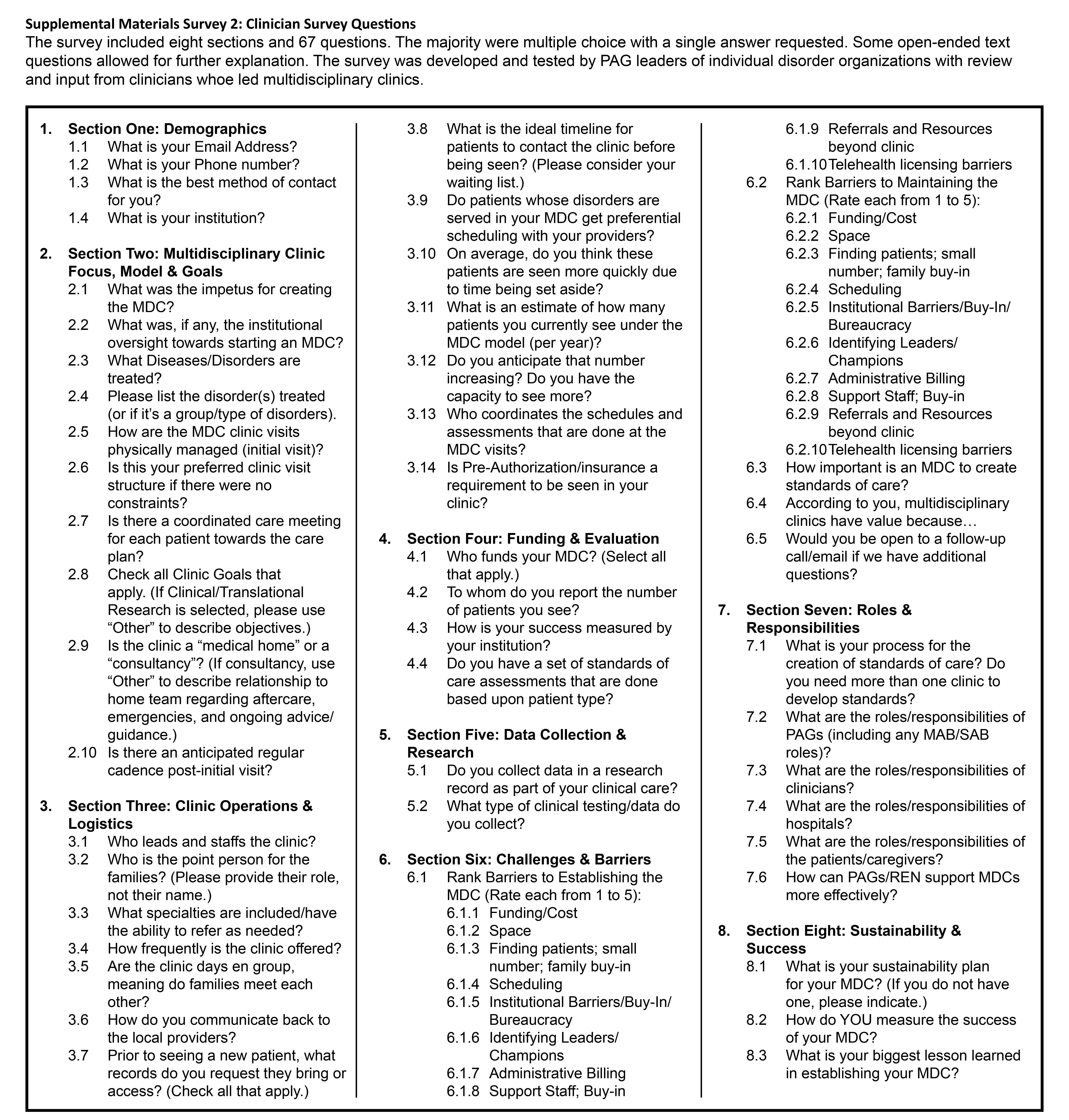

Supplement: Supplementary file 4 [file Supplementary_file_2.jpeg]

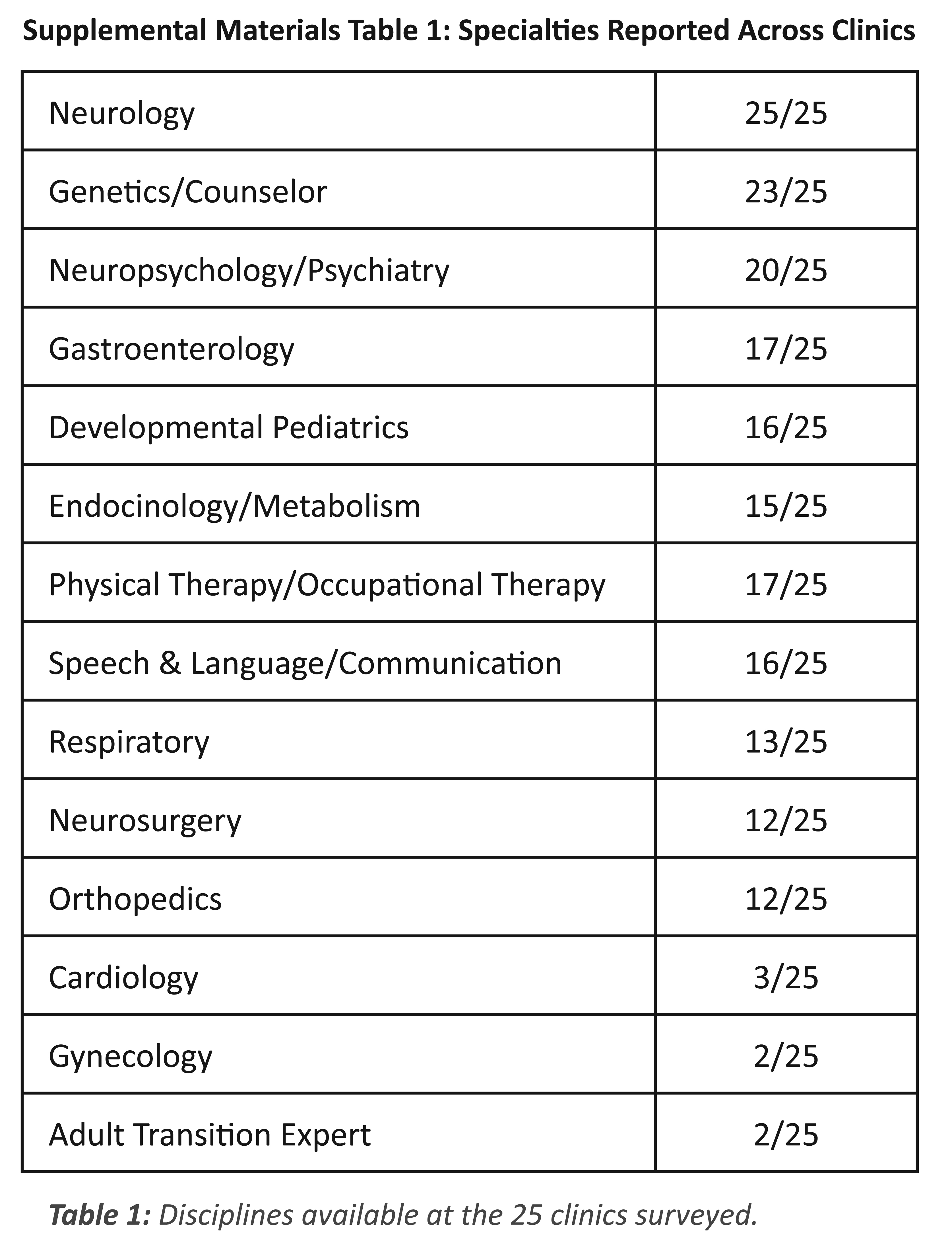

Supplement: Supplementary file 5 [file Supplementary_file_3.jpeg]
